# CRLF1 promotes malignant phenotypes of papillary thyroid carcinoma by activating the MAPK/ERK and PI3K/AKT pathways

**DOI:** 10.1038/s41419-018-0352-0

**Published:** 2018-03-07

**Authors:** Shi-Tong Yu, Qian Zhong, Ren-Hui Chen, Ping Han, Shi-Bing Li, Hua Zhang, Li Yuan, Tian-Liang Xia, Mu-Sheng Zeng, Xiao-Ming Huang

**Affiliations:** 1 Department of Otolaryngology-Head and Neck Surgery, Sun Yat-sen Memorial Hospital, Sun Yat-sen University, 107 Yanjiang West Road, Guangzhou, 510120 China; 2 Guangdong Provincial Key Laboratory of Malignant Tumor Epigenetics and Gene Regulation, Sun Yat-sen Memorial Hospital, Sun Yat-sen University, Guangzhou, 510120 China; 3 State Key Laboratory of Oncology in South China, Sun Yat-sen University Cancer Center, 651 Dongfeng East Road, Guangzhou, 510060 China

## Abstract

Papillary thyroid carcinoma (PTC) is the one of the most common types of endocrine cancer and has a heterogeneous prognosis. Tumors from patients with poor prognosis may differentially express specific genes. Therefore, an analysis of The Cancer Genome Atlas (TCGA) database was performed and revealed that cytokine receptor-like factor 1 (CRLF1) may be a potential novel target for PTC treatment. The objective of the current study was to explore the expression of CRLF1 in PTC and to investigate the main functions and mechanisms of CRLF1 in PTC. PTC tissues exhibited higher CRLF1 expression at both the mRNA and protein levels than it did with normal thyroid tissues. High CRLF1 levels were associated with aggressive clinicopathological features and poor disease-free survival rates. By using loss-of-function and gain-of-function assays, we found that CRLF1 not only increased cell migration and invasion in vitro but also promoted tumor growth both in vitro and in vivo. In addition, CRLF1 induced epithelial–mesenchymal transitions. Overexpression of CRLF1 activated the ERK1/2 and AKT pathways. The oncogenic effects induced by CRLF1 were suppressed by treating the cells with the MEK inhibitor U0126 or the AKT inhibitor MK-2206. These results suggest that CRLF1 enhances cell proliferation and metastasis in PTC and thus may therefore be a potential therapeutic target for PTC.

## Introduction

Papillary thyroid carcinoma (PTC) is the most common cancer of the endocrine system and accounts for most thyroid cancer cases in the past decades^1,2^. PTC is usually indolent and curable and has a 5-year survival rate >95%. However, in some cases, PTC will de-differentiate and become aggressive, resulting in a poor prognosis. Previous reports have revealed that genetic alterations, such as BRAF mutations, TERT mutations, and RET/PTC rearrangement, can promote tumor proliferation and metastasis, which result in poor PTC prognosis; these actions are mediated through the Mitogen-Activated Protein Kinase (MAPK/ERK) and Phosphoinositide 3-kinase (PI3K)/Protein kinase B (AKT) pathways^3^. However, the pathogenesis of PTC has not yet been fully elucidated. Therefore, novel PTC biomarkers must be identified to better predict patients’ prognosis and to facilitate the development of personalized therapies for PTC patients.

Recently, open databases have helped researchers identify cancer-related genes^4^. We analyzed The Cancer Genome Atlas (TCGA) database and found a set of PTC-related candidate genes that were differentially expressed. Among these genes, we found that cytokine receptor-like factor 1 (CRLF1) had a higher fold-change in cancer tissues than in paired normal tissues (average fold-change: 22.54, *P* < 0.001) and a higher fold-change in late-stage cancer tissues than in early-stage cancer tissues (average fold-change: 2.178, *P* < 0.01).

CRLF1 is a secreted protein that belongs to the cytokine receptors family upon assembly with cardiotrophin-like cytokine factor 1 (CLCF1) or p28. It has been reported that CRLF1/CLCF1 or /p28^5–7^ reportedly activates the formation of a specific receptor complex (with ciliary neurotrophic factor receptor, leukemia inhibitory factor receptor-β, and gp130^8,9^) on target cells, thus activating the Janus family kinases, JAK1, 2, and 3, and TYK2^9^, followed by signal transducer and activators of transcription (STAT)^10–13^, particularly STAT3. This process also engages the SH2 domain-containing cytoplasmic protein SHP2 leading to activation of the MAPK/ERK and PI3K/AKT pathways.^9^ CRLF1 can promote normal neuron cells proliferation and survival^5,14^, also sustain B-cell proliferation in mice^15^. Mutations in the CRLF1 gene lead to cold-induced sweating syndrome and Crisponi syndrome^14,16–18^. In addition, some authors have reported that the expression level of CRLF1 was increased in lung adenocarcinoma in human (GDS3627)^19^ compared with that in normal tissues and induced by orthologous in mouse models (GDS1649)^20^. Taken together, these results indicate that CRLF1 may play a significant role in cell survival and proliferation. However, its function in cancer has not yet been investigated. In our study, we found that CRLF1 may serve as a potential biomarker for PTC, and we investigated the potential mechanisms of CRLF1 in PTC.

## Results

### Bioinformatics analysis reveals CRLF1 as a candidate target gene in PTC

**Fig. 1:**
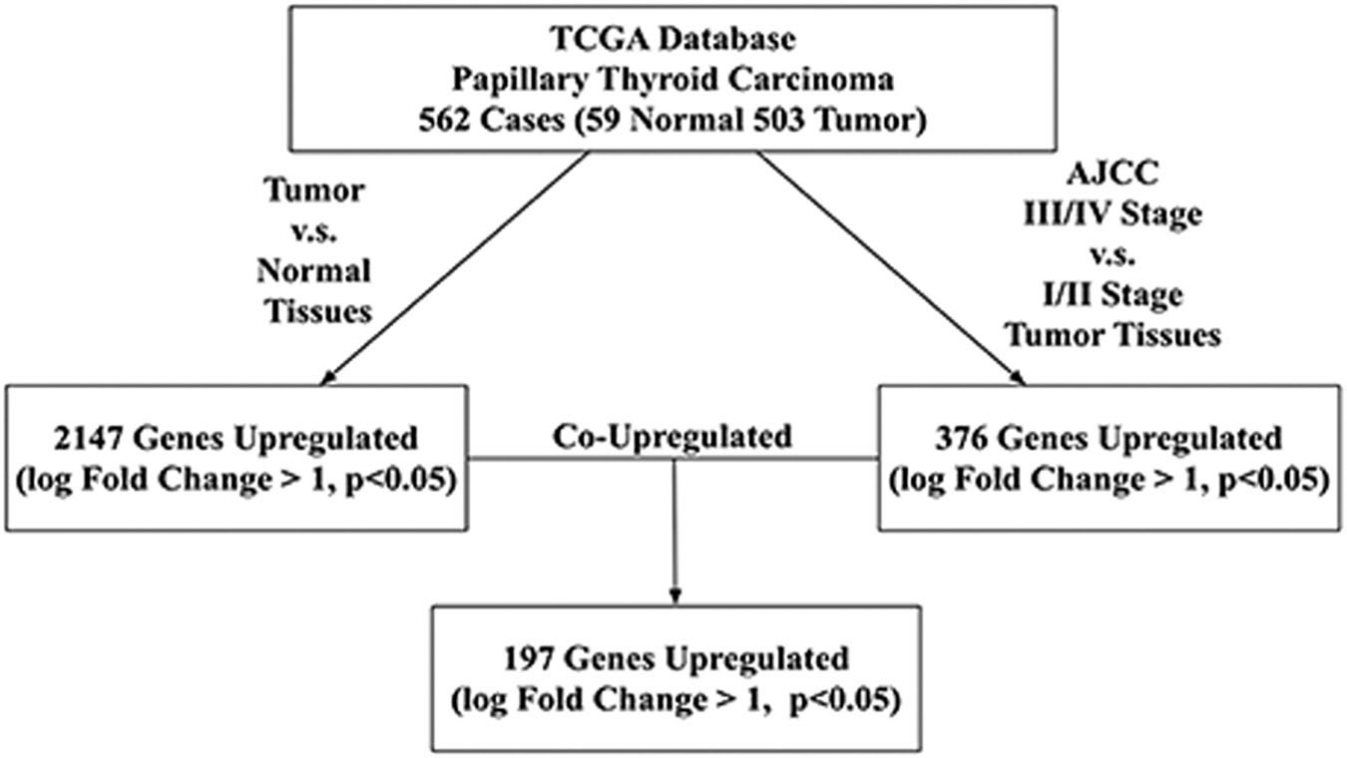
Flowchart for identifying candidate genes from the TCGA database

To identify potential cancer-related genes in PTC, we first analyzed the TCGA database and found a list of 197 cancer-related gene candidates (Fig. 1). Then, we ranked the candidates according to the fold-change ratio of the genes upregulated in cancer tissues compared with those in normal tissues. Among them, most of the top-ranking genes (Supplementary Fig. 1A), including HOXA11, ADRA2C, GRIK3, and PLXNA4, have been extensively studied in various cancers^21–24^. Consequently, we excluded them from this study. After further validation by quantitative reverse transcriptase-PCR (qRT-PCR) using the PTC tissues and corresponding adjacent normal tissues, some other genes (TMEM132A, TREML3, and NXPH4) revealed no significant differences in their expression levels.

**Fig. 2:**
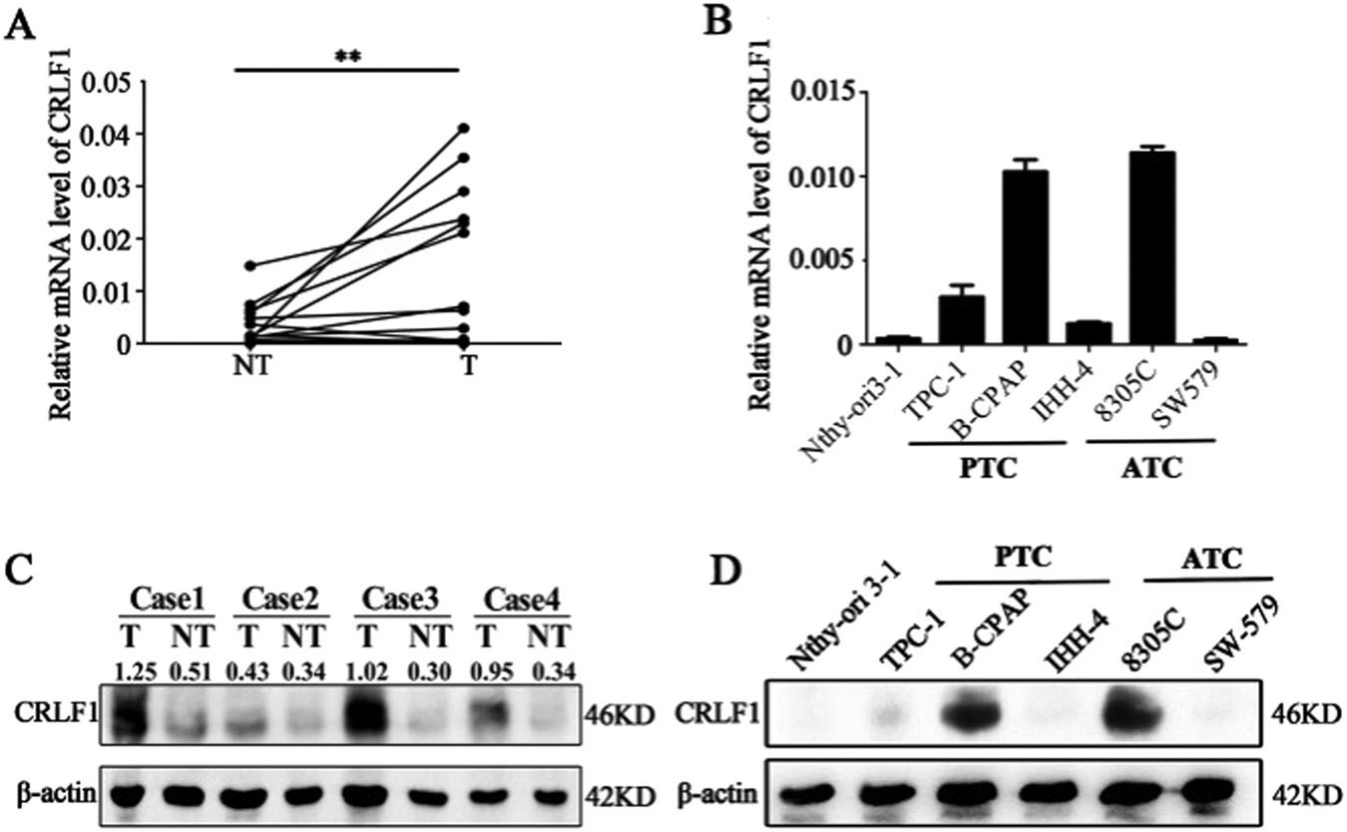
CRLF1 expression in PTC. **a** Expression levels of CRLF1 mRNA in 18 samples of PTC tissue samples (T) were higher than those in their matched normal tissues (NT) according to qRT-PCR (**P < 0.01). **b** mRNA levels of CRLF1 in PTC cell lines (TPC-1, IHH-4 and B-CPAP), anaplastic thyroid carcinoma (ATC) cell lines (8305C and SW579) and an immortalized normal thyroid epithelial cell line (Nthy-ori 3-1) according to qRT-PCR. **c** CRLF1 protein expression levels were higher in PTC tissues (T) than in normal tissues (NT). β-Actin was used as a loading control. **d** CRLF1 protein expression levels in a PTC cell line (B-CPAP) and an ATC cell line (8305C) were higher than those in an immortalized normal thyroid epithelial cell line (Nthy-ori 3-1). β-Actin was used as a loading control

Interestingly, we found that CRLF1 mRNA and protein levels were significantly higher in the PTC tissues than those in the paired normal tissues (Figs. 2a, c). Analysis of the TCGA database revealed that CRLF1 expression was 22.54-fold higher in PTC tissues than that in paired normal tissues (Supplementary Fig. 1B, *P* < 0.001). In addition, CRLF1 expression levels were much higher in classical and tall cell variants of PTC than those in paired normal tissues (Supplementary Fig. 1C, *P* < 0.001), whereas those in follicular variants of PTC were not significantly different. CRLF1 expression levels were higher in patients with lymph node metastasis (N1) than those in patients without lymph node metastasis (N0, *P* < 0.01, Supplementary Fig. 1D). Moreover, CRLF1 expression levels were higher in patients with stage III/IV PTC than those in patients with stage I/II PTC (*P* < 0.01, Supplementary Fig. 1E). We also found that higher CRLF1 expression levels were associated with patients harboring a BRAF V600E mutation (*P* < 0.001, Supplementary Fig. 1F).

To confirm the above results, CRLF1 expression in cell lines was analyzed by qRT-PCR and western blotting assays. Compared with those in a normal epithelial cell line (Nthy-ori-3-1), CRLF1 mRNA and protein levels were increased in a PTC cell line (B-CPAP) and an anaplastic thyroid carcinoma (ATC) cell line (8305C) (Figs. 2b, d). Taken together, these results indicate that CRLF1 is highly expressed in PTC, particularly in patients with lymph node metastasis or in the late stages of the disease.

### High CRLF1 expression levels are associated with aggressive clinicopathological features and poor disease-free survival (DFS) rates

**Fig. 3:**
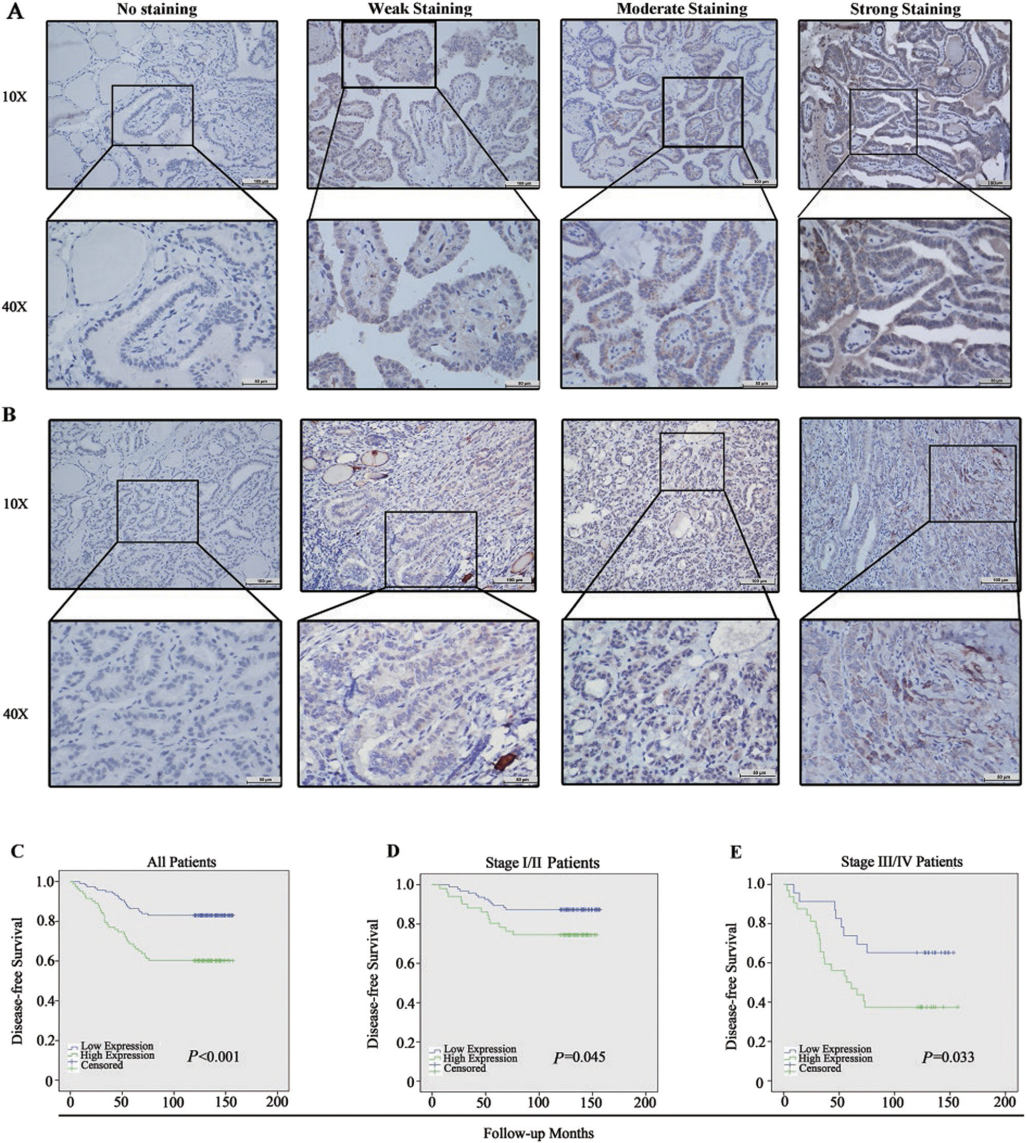
Representative IHC staining for CRLF1 in PTC specimens and Kaplan-Meier estimates of the probability of DFS in PTC patients. CRLF1 was found to be mainly localized in PTC cell cytoplasm. Classical PTC **a** and follicular variant of PTC **b**: no staining, weak staining, moderate staining, and strong staining for CRLF1; the intensity levels were scored as 0, 1, 2, and 3, respectively. **c** A Kaplan–Meier survival analysis shows that high CRLF1 expression levels were significantly associated with poor DFS in all PTC patients (*P* < 0.001). **d** High CRLF1 expression levels are correlated with a shorter DFS in patients with stage I/II (*P* = 0.045) and **e** stage III/IV (*P* = 0.033) PTC

To further investigate the correlation between CRLF1 expression and clinicopathological features, immunohistochemical (IHC) analyses of CRLF1 were performed using 201 paraffin-embedded PTC samples (Fig. 3 and Supplementary Fig. 2). CRLF1 was found to be mainly localized to PTC cell cytoplasm. The cut-off value (2 points) was determined by using the median of the staining index. Tumors with scores of >2 points (83 samples, 41.3%) were considered to have a high CRLF1 expression, and those with scores of ≤2 points were categorized into the group with low CRLF1 expression. Table 1 shows that sex, age, multifocality, M stage, and pathological subtypes were not significantly different among the two groups (*P* > 0.05). However, high CRLF1 expression levels were associated with tumor size, extrathyroidal extension, T stages, N stages, and clinical stages (*P* < 0.05).

**Table 1:** Clinicopathological features of 201 PTC patients

Clinical features	Total	CRLF1 expression		***P***-value
		Low (*n* = 118)	High (*n* = 83)	
Sex				
Male	39	28	11	0.064
Female	162	90	72	
Age (years)				
<45	128	81	47	0.081
≥45	73	37	36	
Extrathyroidal extension				
Yes	70	33	37	**0.015**
No	131	85	46	
Multifocality				
Yes	32	23	9	0.099
No	169	95	32	
T stage				
T1+2	120	80	40	**0.005**
T3+4	81	38	43	
N stage				
N0	102	72	30	**0.001**
N1a+N1b	99	46	53	
M stage				
M0	186	112	74	0.126
M1	15	6	9	
TNM stage				
I+II	146	95	51	**0.003**
III+IV	55	23	32	
Pathological subtype				
Classical	160	90	70	0.162
Follicular	41	28	13	
Tumor size (cm) ± SD	2.09 ± 1.22 cm	1.94 ± 1.22 cm	2.30 ± 1.20 cm	**0.039**

Bold values mean p<0.05

The median follow-up for the 201 PTC patients enrolled in this study was 130 months (range, 3–157 months). Among these patients, 12 patients died due to cancer-related causes, and 53 patients experienced recurrence or persistent disease. A Cox proportional hazards model was carried out to evaluate the prognostic value of CRLF1 expression. Several factors, including extrathyroidal extension, multifocality, T stage, N stage, M stage, tumor-node-metastasis (TNM) stage, and CRLF1 expression, were significantly associated with DFS rates by univariate Cox regression analysis (Table 2). These factors were then further analyzed by multivariate analysis. The multivariate model showed that N stage (hazard ratio (HR) = 3.01, 95% confidence interval (CI) = 1.38–6.57, *P* = 0.006), M stage (HR = 2.28, 95% CI = 1.07–4.83, *P* = 0.032), TNM stage (HR = 1.92, 95% CI = 1.07–3.44, *P* = 0.028), and CRLF1 expression levels (HR = 2.08, 95% CI = 1.18–3.66, *P* = 0.012) were independent indicators of DFS (Table 2).

**Table 2:** Univariate and multivariate COX regression analysis of DFS in relation to clinicopathological features

Clinicopathological features		Univariate analysis		Multivariate analysis	
	All cases	Hazard ratio (95% CI)	*P*-value	Hazard ratio (95% CI)	*P*-value
Sex			0.47		
Male	39	1			
Female	162	0.79 (0.41–1.50)			
Age (years)			0.2		
<45	128	1			
≥45	73	1.42 (0.82–2.44)			
Extrathyroidal extension			**0.002**		0.46
Yes	70	2.38 (1.39–4.08)		0.69 (0.26–1.83)	
No	131	1		1	
Multifocality			0.453		
Yes	32	1.30 (0.65–2.59)			
No	169	1			
T stage			**<0.001**		0.35
T1+2	120	1		1	
T3+4	81	2.80 (1.61–4.89)		1.64 (0.58–4.64)	
N stage			**<0.001**		
N0	102	1		1	**0.006**
N1a+N1b	99	5.47 (2.74–10.89)		3.01 (1.38–6.57)	
M stage			**<0.001**		**0.032**
M0	186	1		1	
M1	15	5.29 (2.72–10.31)		2.28 (1.07–4.83)	
TNM stage			**<0.001**		**0.028**
I+II	146	1		1	
III+IV	55	3.76 (2.20–6.46)		1.92 (1.07–3.44)	
Pathological subtype			0.935		
Classical	160	1			
Follicular	41	1.03 (0.53–2.00)			
CRLF1 expression			**<0.001**		**0.012**
Low	118	1		1	
High	83	2.77 (1.59–4.82)		2.08 (1.18–3.66)	

Bold values mean p<0.05

As shown in Fig. 3b, Kaplan–Meier curves revealed that patients with high expression levels of CRLF1 had a shorter DFS than those with low CRLF1 expression levels (109.8 months vs. 138.0 months, *P* < 0.001). Then, we divided the patients into two subgroups according to their clinical stages (stage I/II and III/IV). We found that the patients with higher CRLF1 expression levels had worse DFS rates in both the stage I/II (*P* = 0.045) and in stage III/IV (*P* = 0.033) groups. Taken together, these results suggest that high expression levels of CRLF1 are correlated with poor survival and that CRLF1 protein expression has significant prognostic value in PTC.

### CRLF1 promotes PTC cell proliferation in vitro and in vivo

**Fig. 4:**
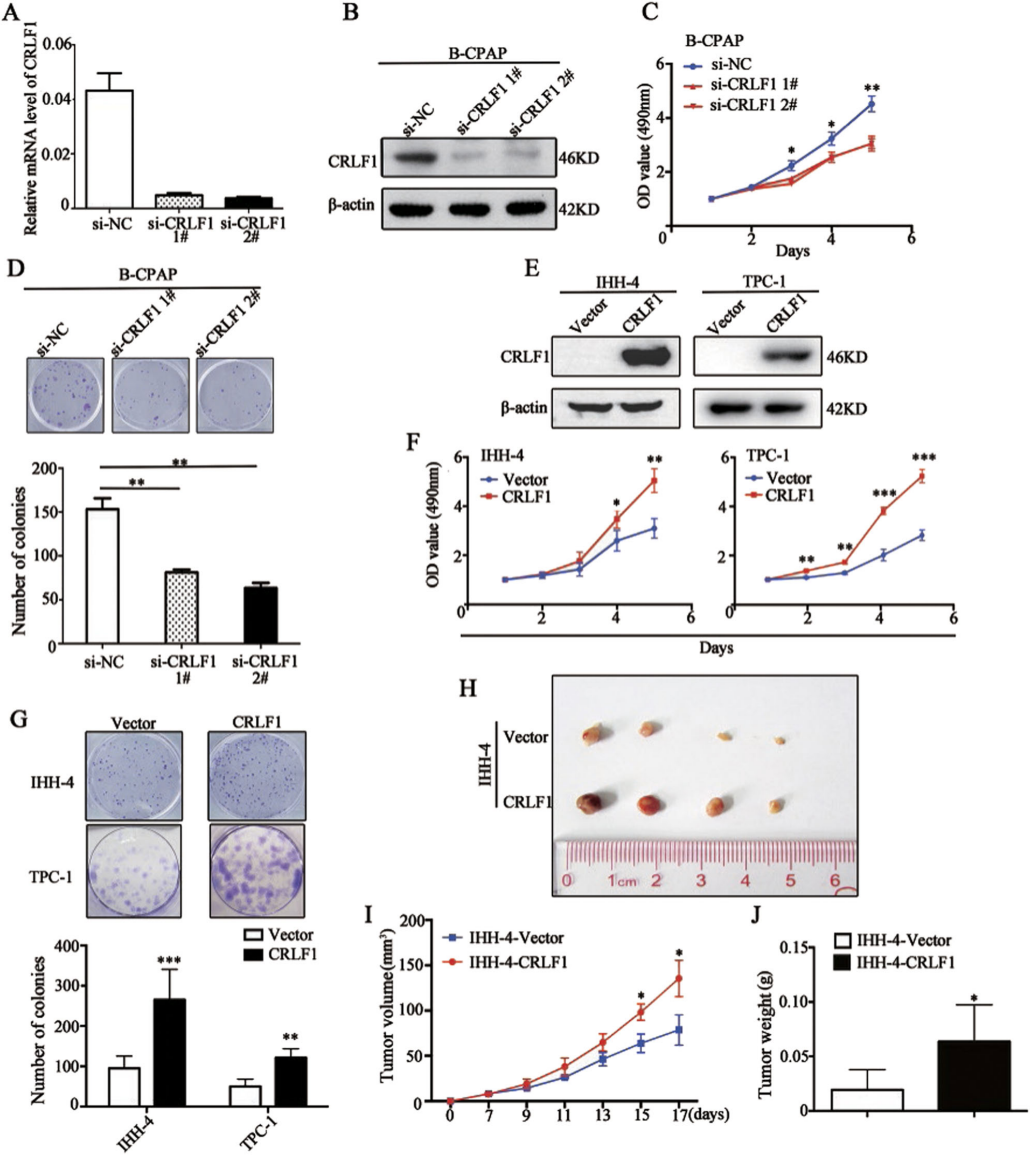
CRLF1 promotes PTC cell growth. Knockdown of CRLF1 mRNA **a** and protein **b** with two different siRNAs (si-CRLF1 1# and 2#) in B-CPAP cells was evidenced by qRT-PCR and western blotting assays, respectively. β-Actin was used for normalization for the qRT-PC assays and as a loading control for the western blotting assays. The data are presented as the mean ± SD. **c** CRLF1 knockdown significantly inhibited cell viability. The data are presented as the mean ± SD. **d** CRLF1 knockdown inhibited the colony formation ability of PTC cells. The upper panel shows representative colony formation images of the cells transfected with the indicated siRNAs. Quantitative analysis of the number of colonies is shown in the lower panel. **e** CRLF1 expression levels were increased after transfection with the CRLF1 expression plasmid in TPC-1 and IHH-4 cells. Ectopic expression of CRLF1 enhanced cell viability **f** and colony formation **g** in TPC-1 and IHH-4 cells. The data are presented as the mean ± SD. **h** Four representative tumors from CRLF1-overexpressing (IHH-4-CRLF1) cells and empty vector-expressing (IHH-4-Vector) cells from nude mice are shown. **i** Tumor growth curves of IHH-4-CRLF1 cells from nude mice are compared with those of vector-control cells. The data are presented as the mean ± SD. **j** Histogram representing the mean tumor weights from the IHH-4-CRLF1 group and the vector-control group. The data are presented as the mean ± ± SD. Significant differences are indicated as follows: *P < 0.05, **P < 0.01 and ***P < 0.001

Gain-of-function and loss-of-function experiments were performed to determine the potential function of CRLF1. As shown in Figs. 4a, b, the expression levels of CRLF1 in the B-CPAP cell line were inhibited by two siRNAs (si-1# and si-2#). The proliferation rate and colony formation ability of B-CPAP cells were lower in the CRLF1 knockdown groups than those in the control small interfering RNA (siRNA) group (si-NC) (Figs. 4c, d).

Furthermore, we overexpressed CRLF1 in both IHH-4 and TPC-1 cell lines, which exhibited low CRLF1 expression (Fig. 4e). Compared with vector transfection, the ectopic expression of CRLF1 in the IHH-4 and TPC-1 cell lines significantly enhanced cell proliferation and colony formation ability (Figs. 4f, g).

Next, we evaluated the effects of CRLF1 on tumor growth in vivo. As shown in Figs. 4h, i, tumors overexpressing CRLF1 in IHH-4 cells grew quicker and had larger volumes than those in the vector group. At the end of the experiments, the xenograft tumors were dissected and weighed. The average tumor weight of the IHH-4-CRLF1 group was significantly higher than that of the vector group (*P* = 0.035, Fig. 4j). Taken together, these results indicate that CRLF1 promotes proliferation both in vitro and in vivo.

### CRLF1 promotes PTC cell migration and invasion and induces the epithelial–mesenchymal transition (EMT)

**Fig. 5:**
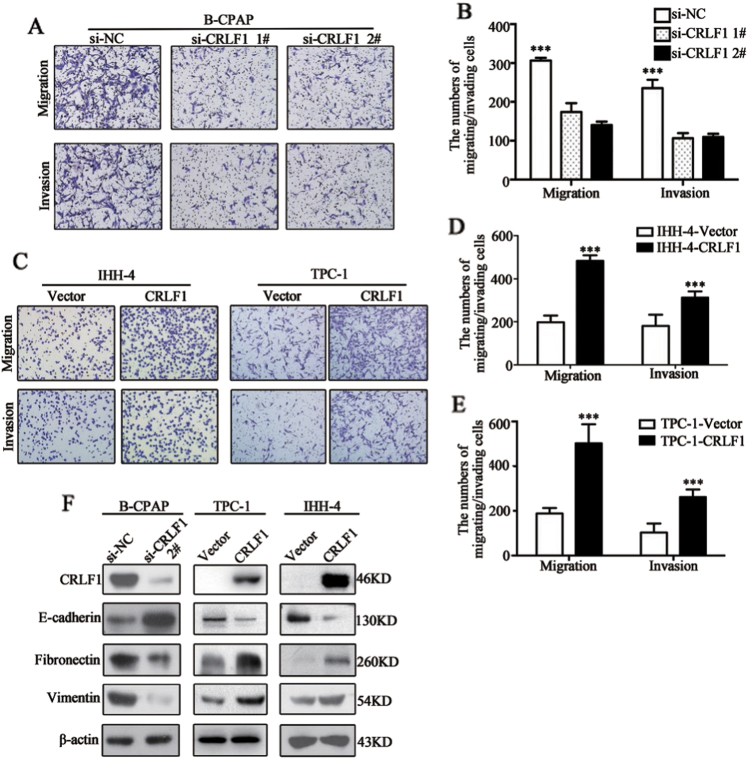
CRLF1 enhances PTC cell migration and invasion and induces the EMT. **a** B-CPAP cells were transfected with two different siRNAs (si-CRLF1 1# and 2#) or si-NC. Representative images of migrating/invading cells are shown. **b** Histograms show the mean ± SD of the number of migrating/invading cells from three independent assays. **c** Representative images of migrating/invading TPC-1 and IHH-4 cells expressing the empty vector or the CRLF1 plasmid. **d** Histograms show the mean ± SD of the number of migrating/invading cells from three independent assays. **f** Protein levels of EMT markers that changed with CRLF1 knockdown or CRLF1-overexpressing cell lines. β-Actin was used as a loading control. Significant differences are indicated as follows: *** *P* < 0.001

According to the above results showing that high CRLF1 expression levels were correlated with the N and M stages, we investigated whether CRLF1 influenced the migration and invasion abilities of B-CPAP cells. As shown in Figs. 5a, b, the number of migrated cells transfected with si-CRLF1-1# or si-CRLF1-2# was significantly lower than the number of migrated cells transfected with si-NC. Similarly, invasion assays showed that knocking down CRLF1 via siRNAs resulted in fewer cells invading through the Matrigel-coated membrane. Moreover, the ectopic expression of CRLF1 in IHH-4 and TPC-1 cells promoted migration and invasion (Figs. 5c–e). These results indicate that CRLF1 enhances the migration and invasion abilities of PTC cells.

Recent reports have shown that the EMT is associated with cancer metastasis^25–27^. Therefore, we examined the association between EMT markers and CRLF1 expression. As shown in Fig. 5f, levels of the epithelial marker E-cadherin were decreased, and the levels of mesenchymal markers, including vimentin and fibronectin, were increased in CRLF1-overexpressing cells compared with those in vector-expressing cells. As expected, E-cadherin was upregulated; however, fibronectin and vimentin were downregulated in CRLF1 knockdown cells.

### CRLF1 regulates tumorigenesis through the MAPK/ERK and PI3K/AKT signaling pathways

**Fig. 6:**
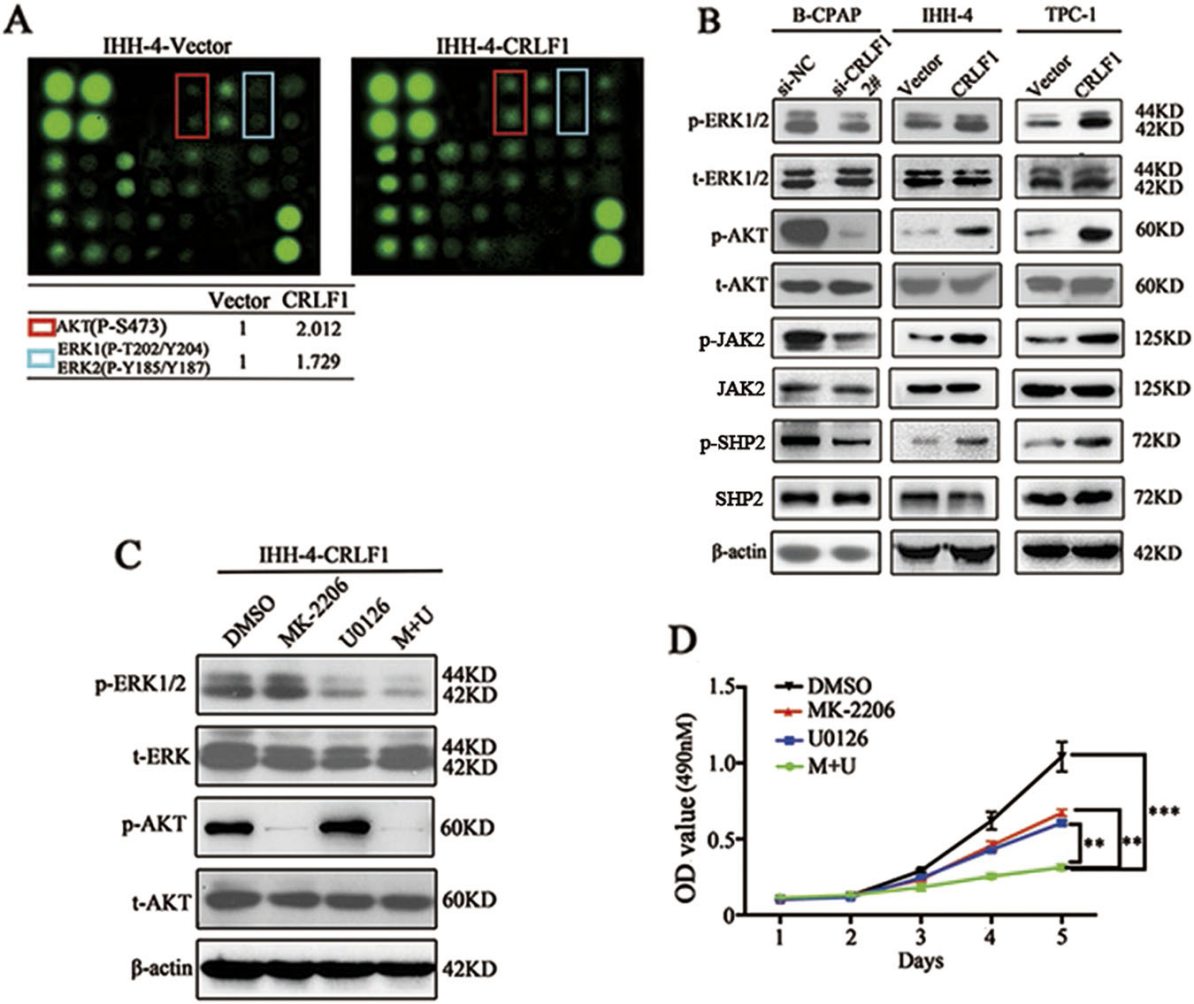
CRLF1 promotes tumorigenesis by activating the MAPK/ERK and PI3K/AKT pathways. **a** An MAPK phosphorylation antibody array revealed that ERK1/2 and AKT (P-S473) were activated in CRLF1-overexpressing IHH-4 cells. **b** Western blotting assays showed changes in the levels of ERK1/2 and AKT (P-S473) in CRLF1 knockdown or CRLF1-overexpressing cells. **c** IHH-4-CRLF1 cells were cultured with 10 μM U0126, 10 μM MK-2206, or 10 μM U0126+10 μM MK-2206 for 24 h. Western blotting analyses were performed to evaluate the effects of these two inhibitors on phosphorylation levels of ERK1/2 and AKT. β-Actin was used as a loading control. **d** MTT proliferation assay results demonstrating inhibited proliferation in CRLF1-overexpressing IHH-4 cells treated with MK-2206, U0126, or MK-2206+U0126

To determine the underlying mechanism by which CRLF1 affects tumorigenesis in PTC cells, signaling pathways were assessed in CRLF1-overexpressing and empty vector-expressing IHH-4 cells using an intercellular signaling array. The phosphorylation levels of AKT (p-S473) and ERK1/2 (p-T202/Y204, p-Y185/Y187) were higher in CRLF1-overexpressing cells than those in the controlled cells (Fig. 6a, Supplementary Fig. 3). These results were consistent with previous reports^5,6,15,28^. Then, we further analyzed intracellular effectors and relevant signaling pathways by western blotting in both CRLF1 knockdown and CRLF1-overexpressing PTC cells. We found that CRLF1 may activate JAK2 and SHP2 phosphorylation, thus activating ERK1/2 and AKT (p-S473) phosphorylation. The total levels of ERK and AKT remained unchanged (Fig. 6b). Additionally, greater levels of p-ERK1/2 and p-AKT were detected in tumors generated from CRLF1-overexpressing IHH-4 cells than those in tumors generated from control cells (Supplementary Fig. 4). STAT3 phosphorylation level were higher in IHH-4-CRLF1 cells than those in IHH-4-Vector cells (Supplementary Fig. 5A). Then, U0126 (an MEK inhibitor) or MK-2206 (an AKT inhibitor) was added to evaluate whether these inhibitors could affect the growth rate of CRLF1-overexpressing IHH-4 cells. As expected, the phosphorylation levels of AKT and ERK but not the total levels of ERK and AKT, were downregulated after the treatment with either U0126 or MK-2206 (Fig. 6c). Figure 6d shows that both U0126 and MK-2206 could reduce the growth rate. Combining the two inhibitors significantly decreased the growth rate compared with using either inhibitor alone. However, treatment of Stattic (a STAT3 inhibitor) resulted in no change in the growth rate (Supplementary Fig. 5B and 5C). Taken together, these data indicate that CRLF1 may regulate tumorigenesis, at least in part through the MAPK/ERK and PI3K/AKT signaling pathways.

## Discussion

In this study, we showed evidence supporting the oncogenic effects of CRLF1 in PTC. First, CRLF1 expression was higher in PTC tumor tissues than that in matched non-tumor tissues. Second, patients with high CRLF1 expression levels had aggressive clinicopathological features and poor clinical outcomes. Third, CRLF1 may regulate the MAPK/ERK and PI3K/AKT pathways to contribute to PTC tumorigenesis.

CRLF1 has been reported to stimulate growth and survival in neurons^29^. However, its function in various types of cancer, especially in PTC, remains unclear. Therefore, we performed in vitro and in vivo experiments to explore its biological function. Our data showed that ectopic expression of CRLF1 significantly increased cell growth and colony formation ability in vitro and induced tumor formation in nude mice in vivo. Furthermore, the EMT is known to be associated with invasion and metastasis in various cancers, including PTC^30–34^. Therefore, we evaluated whether CRLF1 affected PTC cell migration and invasion and the EMT. As expected, CRLF1-overexpressing cells showed high rates of migration and invasion. We also found that E-cadherin was downregulated in CRLF1-overexpressing cells. In addition, CRLF1 increased the levels of the mesenchymal markers vimentin and fibronectin. These findings demonstrate that CRLF1 induces the EMT in PTC cells. In contrast, CRLF1 knockdown inhibited cell growth and invasion, further supporting that CRLF1 may promote PTC cell malignant phenotype.

The MAPK/ERK and PI3K/AKT pathways have been reported to play an important role in PTC tumorigenesis^3,35–38^. Previous reports have suggested that CRLF1 can promote intracellular effectors following activation of the MAPK/ERK, PI3K/AKT, and JAK/STAT pathways by forming specific receptor complexes with CLCF1 or p28 on target cells^5,6,15,29^. Additionally, CRLF1/CLCF1 enhances motor neurons survival in vitro^5,7,39^ and in vivo^7^. We performed pull-down assay and mass spectrometry assay to verify whether CRLF1 could bind p28 or CLCF1 in PTC cells and thus trigger activation of these pathways. However, neither CLCF1 nor p28 were found to bind directly with CRLF1 (data not shown), possibly due to the limited sensitivity of the pull-down assay and mass spectrometry assay or other potential mechanisms involved in CRLF1-induced tumorigenesis. However, our data showed that CRLF1 activated intracellular effectors including JAK2 and SHP2 and thus increased the phosphorylation levels of ERK1/2 and AKT in vitro. Moreover, treatment with the MEK inhibitor U0126, the AKT inhibitor MK-2206, or a combination of both blocked the effects induced by CRLF1. However, blockage of STAT3 resulted in no change in the growth rate. The role of STAT3 in thyroid cancer tumorigenesis is still inconclusive^40–43^, indicating that the underlying mechanism should be investigated in the future. Therefore, the MAPK/ERK and PI3K/AKT signaling pathways may be involved in CRLF1-induced tumorigenesis in PTC. Taken together, our data show that CRLF1-overexpressing PTC cells may activate MAPK/ERK and PI3K/AKT signaling, indicating that CRLF1 may be a possible therapeutic target for PTC treatment.

Although we found that CRLF1 may induce PTC cells malignant phenotype by activating the MAPK/ERK and PI3K/AKT signaling pathways, there are several limitations in this study. First, the IHC patient cohort included a relatively small number of patients and a short follow-up period. Therefore, a larger cohort of patients and a longer follow-up period should be used to verify these results in the future. Second, the underlying mechanism of how CRLF1 triggers the MAPK/ERK and PI3K/AKT pathways to induce PTC tumorigenesis remains unclear. Further studies on this mechanism are warranted.

In summary, for the first time, we have shown that CRLF1 is upregulated in human PTC tissues and that its expression is associated with aggressive clinicopathological features and a poor prognosis. Moreover, our data suggest that CRLF1 plays an oncogenic role in PTC tumorigenesis by regulating the MAPK/ERK and PI3K/AKT signaling pathways. These results indicate that CRLF1 is a potential biomarker in PTC patients and that it may be a valuable therapeutic target for PTC in the future.

## Materials and methods

### Analysis of the TCGA database and verification of cancer-related candidate genes

The clinical information and genomic data for 507 PTC (THCA) samples (Level 2) were retrieved from the TCGA database (http://cancergenome.nih.gov/) in November 2015. All mRNA expression levels of the samples were normalized and measured using the Illumina HiSeq V2 platform. The protocol for screening cancer-related candidate genes was as follows (Fig. 1a). First, a group of genes that are differentially expressed in cancer and normal tissues was selected (cancer tissue overexpression of a log fold-change >1, *P* < 0.05). Then, another group of genes that are differentially expressed in stage III/IV and stage I/II cancer tissues was selected (stage III/IV overexpression of a log fold-change >1, *P* < 0.05). The genes overexpressed in both of these groups were considered candidate genes. Ultimately, the expression of 18 pairs of complementary DNA from cancer tissues and paired normal tissues was verified by qRT-PCR.

### Patients and clinical tissue samples

Written informed consents was received from all patients before enrollment, and this study was approved by the Ethics Committee of Sun Yat-sen Memorial Hospital, Sun Yat-sen University. Sixteen PTC tissue samples and their paired normal tissue samples were obtained in June 2016 for qRT-PCR and western blotting analyses. A total of 201 paraffin-embedded PTC samples, from patients who were first diagnosed between January 2003 and December 2006 at Sun Yat-sen Memorial Hospital, Sun Yat-sen University, were collected for IHC analyses. All medical histories of the patients were well-documented according to 7th Edition of the American Joint Committee on Cancer (AJCC) TNM system. These samples were obtained from 39 men and 162 women with a median age of 41 years (range, 14–74). All patients were followed up every 3–5 months during the first 5 years and then every year thereafter. Recurrence/persistent disease referred to recurrent or persistent disease with either an incomplete biochemical response or an incomplete structural response^44^. Patients with suppressed thyroglobulin (Tg) levels >1 ng/mL, thyroid-stimulating hormone (TSH)-stimulated Tg levels >10 ng/mL, or increased anti-Tg antibody levels in the absence of structural disease were defined as having an incomplete biochemical response^44^. Patients with proven histology/cytology results or suspicious lesions according to imaging studies were defined as having structural disease^44^. DFS was defined as the time from the date of surgery to the date of relapse, metastasis, or the last follow-up. All patients’ survival statuses were confirmed in December 2016.

### IHC analysis

Clinical PTC tissue samples and tumors resected from mice were embedded in paraffin. Briefly, 4-m-thick sections were cut and baked at 60 °C for 2 h. Then, the sections were deparaffinized with xylene and rehydrated, and the endogenous peroxidase activity was blocked with 0.3% H_2_O_2_. Next, the sections were processed for high-temperature antigen retrieval with citrate (pH 6.0) and incubated with 5% bovine serum albumin to block nonspecific binding. The sections were then incubated with diluted rabbit anti-CRLF1 antibody (1:100; HPA041493, Sigma-Aldrich, USA), p-ERK1/2 antibody (1:400; #4370, Cell Signaling Technology, USA), or p-AKT antibody (1:100; #4060, Cell Signaling Technology, USA) at 4 °C overnight. Next, these slides were washed three times with phosphate-buffered saline plus 1:1000 Tween-20 and incubated with secondary antibodies (1:1000) for 30 min at 37 °C. The sides were immersed in diaminobenzidine (Zhongshan Biological and Technical Company, Beijing, China) for 10 min, and the reaction was terminated with distilled water. Then, the slides were counterstained with hematoxylin, dehydrated and cover slipped. All sections were scored by two experienced pathologists. The staining index of CRLF1 was calculated as follows: staining index = staining × intensity proportion of positive tumor cells. Staining intensity was defined as follows: 0 (no staining); 1 (weak, light yellow); 2 (moderate, yellow-brown); and 3 (strong, brown). The percentage of positive cells was defined as follows: 0 (no positive cells); 1 (<10% positive tumor cells); 2 (10–50% positive tumor cells); and 3 (>50% positive tumor cells). The staining index cut-off value for CRLF1 expression was determined by using its median value (2 points). A staining index score of >2 points was used to define tumors with high expression, and a staining index score of 2 points was used to defined tumors with low expression.

### Western blotting assay

Total protein was lysed in one sodium dodecyl sulfate (SDS) sample buffer and protein concentrations were measured by BCA protein assays. Protein extracts were separated on 8–12% SDS-polyacrylamide gels by electrophoresis, transferred to polyvinylidene fluoride membranes (Millipore, USA), and blocked with 5% skim milk or bovine serum albumin for 1 h. Then, the membranes were incubated with primary antibodies at 4 °C overnight and with horseradish peroxidase-conjugated secondary antibodies (Pierce, USA) at room temperature for 1 h. Next, bound antibodies were visualized via enhanced chemiluminescence and captured using XAR film. β-Actin was used as a loading control. The primary antibodies used in this study were as follows: human anti-CRLF1 (1:400; ab56500, Abcam, USA), anti-E-cadherin (1:1000; #14472, Cell Signaling Technology, USA), anti-fibronectin (1:1000; ab32419, Abcam, USA), anti-vimentin (1:1000; ab8978, Abcam, USA), anti-ERK1/2 (1:1000; #4695, Cell Signaling Technology, USA), anti-p-ERK1/2 (1:1,000; #4370, Cell Signaling Technology, USA), anti-AKT (1:1000; #9272, Cell Signaling Technology, USA), anti-p-AKT (S473, 1:1000; #4060, Cell Signaling Technology, USA), anti-STAT3 (1:1000; #9193, Cell Signaling Technology, USA), anti-p-STAT3 (Tyr705,1:1000; #9145, Cell Signaling Technology, USA), anti-SHP2 (1:1000; #3397, Cell Signaling Technology, USA), anti-p-SHP2 (1:1000; #3751, Cell Signaling Technology, USA), anti-JAK2 (1:1000; #3230, Cell Signaling Technology, USA), anti-p-JAK2 (1:1000; #3771, Cell Signaling Technology, USA), and β-actin (1:4,000, Sigma-Aldrich, A5541, USA).

### qRT-PCR assay

TRIzol Reagent (Invitrogen, USA) was used to isolate total RNA from PTC cells and clinical tissues. Then, 2 μg of RNA was reverse-transcribed using M-MLV Reverse Transcriptase (Promega). The threshold cycle value of each sample was assessed by qRT-PCR using SYBR Green (Invitrogen) and a CFX96 Touch sequence detection system (Bio-Rad, USA). β-Actin was used as an internal control for all genes. The relative gene expression levels were calculated using the comparative threshold cycle (2^−ΔΔCT^) equation. All experiments were run independently in triplicate, and the sequences of the primers were as follows:

CRLF1 sense 5′-GGGATCTGGAGTGAGTGGAGC-3′;

anti-sense 5′-GGGTCTTGTGCGACTTCTGC-3′;

β-actin sense 5′- CGCGAGAAGATGACCCAGAT-3′;

anti-sense 5′-GGGCATACCCCTCGTAGATG-3′.

### Cell lines and cell culture

Human PTC IHH-4, B-CPAP, and normal thyroid epithelial Nthy-ori-3-1 cell lines were gifts from Haixia Guan (The First Affiliated Hospital of China Medical University, Shenyang, China). The human PTC cell line TPC-1 was purchased from Nanjing Cobioer Company (Cobioer, China). Human ATC 8305C cell line was purchased from the European Collection of Cell Culture (ECACC, Salisbury, UK). The human ATC SW579 and the human embryonic kidney 293T (293T) cell lines were purchased form the American Type Culture Collection (ATCC, Manassas, VA, USA). The IHH-4 cell line was cultured in RPMI-1640 and Dulbecco’s modified Eagle’s medium (DMEM; Invitrogen) supplemented with 10% fetal bovine serum (FBS, Gibco, USA). The Nthy-ori-3-1 and B-CPAP cell lines were cultured in RPMI-1640 supplemented with 10% FBS; The 8305C, SW579, TPC-1, and 293T cell lines were cultured in DMEM supplemented with 10% FBS. All cell lines were cultured with penicillin (100 U/mL) and streptomycin (100 U/mL) at 37 °C in a humidified 5% CO_2_ incubator.

### RNA interference and plasmid transfection

Effective siRNA oligonucleotides that targeting CRLF1 were purchased from Guangzhou Ribobio Company (Guangzhou, China) and were transfected using Lipofectamine RNAiMax (Invitrogen) according to the manufacturer’s instructions. The lentiviral vector encoding FLAG-tagged CRLF1 (EX-N0027-Lv121), the control vector (EX-EGFP-Lv105), and the packaging system (HIV) were obtained from GeneCopeia (USA). All the plasmids were verified by DNA sequencing. The siRNA sequences used were as follows:

siRNA 1# of CRLF1 sense 5′-GGCUCUCUUACGCCCUAU dTdT-3′;

anti-sense 5′-AUAGGGCGUAAAGAGAGCC dTdT-3′;

siRNA 2# of CRLF1 sense 5′-CACGCUGGAUAUCCUGGAU dTdT-3′;

anti-sense 5′-GUGCGACCUAUAGGACCUA dTdT-3′.

The lentivirus packaging expression plasmids were co-transfected into 293T cells. Then, the supernatants containing viruses were collected and used to infect the PTC cell lines for 48 h. Then, stable clones of these cells were selected with puromycin (Sigma-Aldrich, USA) for 7 days after infection. The expression levels of CRLF1 were verified by qRT-PCR and western blotting assays.

### MTT and colony formation assays

A total of 800–1200 cells in 200 μL of medium were seeded per well in 96-well plates (five replicates of each sample). Next, 20 μL of MTT (5 mg/mL, BD Biosciences) was added per well on the indicated day (days 1, 2, 3, 4, or 5) and incubated for 4 h at 37 °C.Then, the supernatants were discarded, and 100 μL of dimethylsulfoxide (DMSO) was added per well to dissolve the crystals. A spectrophotometric plate reader (BioTek ELX 800, USA) was used to measure the absorbance at 490 nm.

For the colony formation assays, 800 cells in 2 mL of medium per well were seeded into six-well plates and cultured for 7–10 days. The colonies were fixed with methanol for 10 min and stained with 0.5% crystal violet for 15 min. Each experiment was performed in three times.

### Transwell migration and invasion assay

Transwell chambers (8 μm pores, Corning, USA) were used for the cell migration and invasion assays. They were pre-coated without (migration assay) or with (invasion assay) Matrigel (BD Biosciences). First, 5×10^4^–1×10^5^ cells suspended in 200 μL of serum-free medium were seeded in the upper chambers, and 600 μL of medium supplemented with 10% FBS was plated in the lower chambers. After 24–36 h of incubation, the cells on the upper surface of the membrane were fixed with methanol for 10 min and stained with 0.5% crystal violet for 15 min. Then, an inverted microscope was used to count the cell numbers.

### Phosphorylation antibody array

IHH-4-Vector/CRLF1 (5×10^5^) cells were plated in 10-cm dishes. Cell lysates were collected after the cells reached 80% confluence and were analyzed with a commercial MAPK phosphorylation antibody array (RayBiotech, Norcross, GA, USA) according to the manufacturer’s instructions. Briefly, the membranes were blocked with blocking buffer for 30 min at room temperature and incubated with 2 mL of the supernatants (diluted 1:2 in blocking buffer) overnight at 4 °C. After washing, a biotin-conjugated antibody detection cocktail was added for incubation overnight at 4 °C, followed by an additional overnight incubation at 4 °C with streptavidin-conjugated peroxidase at room temperature. The membranes were incubated with peroxidase substrate, and the results were documented using XAR films. Chemiluminescence signaling intensity was quantified using Quantity One software (Bio-Rad).

### Drug treatments

CRLF1-overexpressing IHH-4 cells were grown as described above until they reached 40% confluence. Then, the media were replaced with RPMI-1640 containing vehicle (0.1% (v/v) DMSO), U0126 (10 μM) (S1102, Selleck, Houston, TX), MK-2206 (10 μM) (S1078, Selleck, Houston, TX), or U+M (10 *μ*M U0126+10 *μ*M MK-2206). Samples were collected at different time intervals as indicated for each experiment.

### Animal studies

All animal experiments were approved by the Institutional Research Medical Ethics Committee of Sun Yat-sen Cancer Center. Four- to 6-week-old female BALB/c nude mice (*n* = 8) were purchased from Beijing Vital River Laboratory Animal Technology Co., Ltd. (Beijing, China) and randomly divided into two groups (four mice per group). Tumor xenografts were established by subcutaneously injecting 100 μL of a mixture containing 70% vector or CRLF1-expressing IHH-4 cells (5 × 10^6^) and 30% Matrigel. Tumor sizes were measured every 2 days, and tumor volumes were calculated using the following equation: 0.5×length×width^2^. After 17 days, the mice were sacrificed, and the tumors were harvested, weighted, and embedded in 10% paraffin. Each tissue was subjected to analyze the express of markers (CRLF1, p-ERK1/2, and p-AKT) by IHC, as described previously.

### Statistics analysis

All data were analyzed using SPSS Ver.22.0 (IBM Corporation, USA) and GraphPad Prism Ver.7.0 (GraphPad Software, San Diego, CA, USA). All data are shown as the mean ± SD and were obtained from three independent experiments;^2^ or Fisher exact tests were used for categorical variables. Comparisons between two groups were assessed by Student’s *t*-tests. Log-rank tests were used to estimate differences in survival rates among different groups. In addition, the Kaplan–Meier method was used to estimate survival curves. Multivariate Cox regression analyses were performed to determine independent prognostic factors based on the factors that were significant in the univariate Cox regression analyses. A two-tailed *P*-value of < 0.05 was considered to be statistically significant. *Indicates *P* < 0.05, ** indicates *P* < 0.01, and *** indicates *P* < 0.001.

## Electronic supplementary material

Supplementary Figure 1

Supplementary Figure 2

Supplementary Figure 3

Supplementary Figure 4

Supplementary Figure

Supplementary Figure Legends
