# Supplementary material for: CRLF1 promotes malignant phenotypes of papillary thyroid carcinoma by activating the MAPK/ERK and PI3K/AKT pathways
Source: Cell Death Dis. 2018 Mar 7;9(3):371. doi: 10.1038/s41419-018-0352-0 (PMC5841418; doi:10.1038/s41419-018-0352-0)

A

Ranking	Gene Name	Log Fold Change	Ratio of Upregulation Compared to Normal Tissues
1	HOXA11	4.533853634	0.882352941
2	TMEM132A	1.769596758	0.882352941
3	ADRA2C	2.269786735	0.862745098
4	TREML3	2.961794728	0.843137255
5	GRIK3	2.928952261	0.803921569
6	NXPH4	3.238465673	0.803921569
7	PLXNA4	2.040243623	0.803921569
8	CRLF1	4.494155322	0.784313725

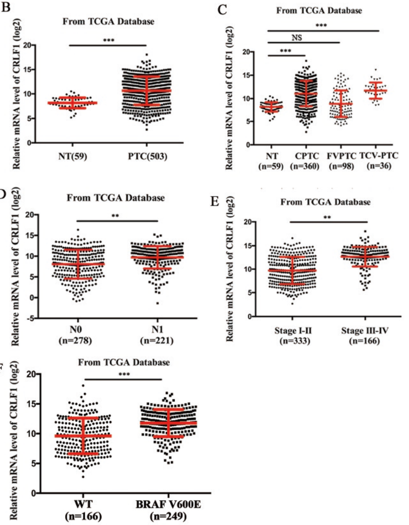

Supplement: Supplementary file 1 — Supplementary Figure 1 [file 41419_2018_352_MOESM1_ESM.pdf]
