# Supplementary figures and images for: CRLF1 promotes malignant phenotypes of papillary thyroid carcinoma by activating the MAPK/ERK and PI3K/AKT pathways
Source: Cell Death Dis. 2018 Mar 7;9(3):371. doi: 10.1038/s41419-018-0352-0 (PMC5841418; doi:10.1038/s41419-018-0352-0)

**A****10X**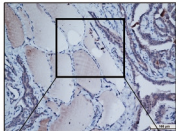**40X**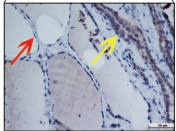**B**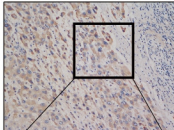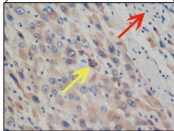**C**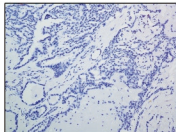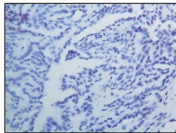

Supplement: Supplementary file 2 — Supplementary Figure 2 [file 41419_2018_352_MOESM2_ESM.pdf]

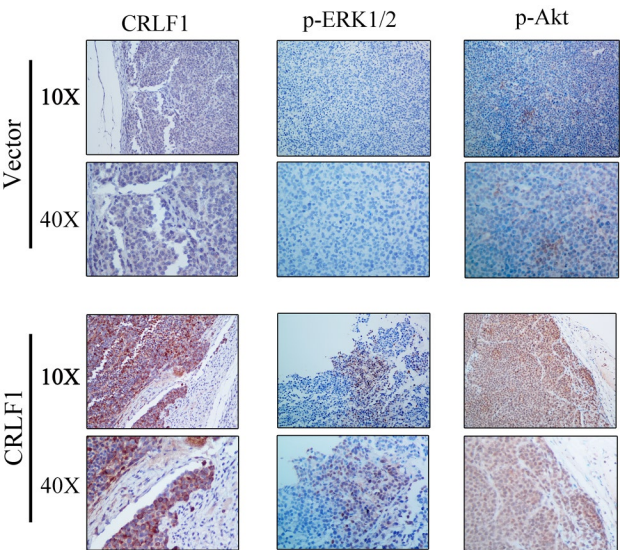

Supplement: Supplementary file 4 — Supplementary Figure 4 [file 41419_2018_352_MOESM4_ESM.pdf]

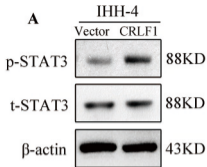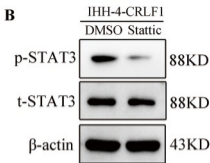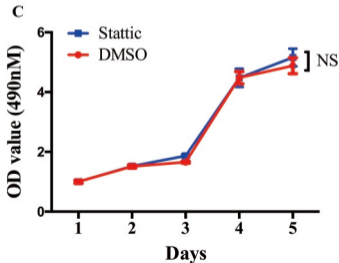

Supplement: Supplementary file 5 — Supplementary Figure [file 41419_2018_352_MOESM5_ESM.pdf]
