# Supplementary material for: CRLF1 promotes malignant phenotypes of papillary thyroid carcinoma by activating the MAPK/ERK and PI3K/AKT pathways
Source: Cell Death Dis. 2018 Mar 7;9(3):371. doi: 10.1038/s41419-018-0352-0 (PMC5841418; doi:10.1038/s41419-018-0352-0)
Supplement: Supplementary file 3 — Supplementary Figure 3 [file 41419_2018_352_MOESM3_ESM.pdf]

A

Each antibody is spotted in duplicate vertically

	A	B	C	D	E	F	G	H
1, 2	POS	POS	NEG	NEG	Akt (P-S473)	CREB (P-S133)	ERK1 (P-T202/Y204) ERK2 (P-Y185/Y187)	GSK3a (P-S21)
3, 4	GSK3b (P-S9)	HSP27 (P-S82)	JNK (P-T183)	MEK (P-S217/221)	MKK3 (P-S189)	MKK6 (P-S207)	MSK2 (P-S360)	mTOR (P-S2448)
5, 6	p38 (P-T180/Y182)	P53 (P-S15)	P70S6K (P-T421/S424)	RSK1 (P-S380)	RSK2 (P-S386)	NEG	NEG	POS

B

Upregulated Markers	Fold Change
MSK2	1.517
P53	1.560
GSK3a	1.593
mTOR	1.648
P70S6k	1.648
HSP27	1.650
GSK3b	1.752
P38	1.760
MKK3	1.771
RSK2	2.343
