# Supplementary material for: CRLF1 promotes malignant phenotypes of papillary thyroid carcinoma by activating the MAPK/ERK and PI3K/AKT pathways
Source: Cell Death Dis. 2018 Mar 7;9(3):371. doi: 10.1038/s41419-018-0352-0 (PMC5841418; doi:10.1038/s41419-018-0352-0)
Supplement: Supplementary file 6 — Supplementary Figure Legends [file 41419_2018_352_MOESM6_ESM.docx]

**Supplementary Figure Legends**

**Supplementary Fig. 1** (A) List of candidate genes selected from TCGA database. In the dataset from the TCGA database, (B) CRLF1 expression was higher in PTC tissues than that in normal thyroid tissues (NT). (C) Classical PTC (CPTC) and tall cell variant PTC (TCV-PTC) tissues, but not follicular variant PTC (FV-PTC) tissues, had higher CRLF1 expression levels than those in normal thyroid tissues (NT). (D) Patients with lymph node metastasis (N1) had higher CRLF1 levels than those in patients without lymph node metastasis (N0). (E) CRLF1 expression levels in patients with stage III/IV PTC were higher than those in patients with stage I/II PTC. (F) CRLF1 expression level in patients with the BRAF V600E mutation were higher than those in BRAF-wild type patients. Horizontal lines indicate the median and interquartile range. Significant differences are indicated as follows: ** *P*<0.01 and *** *P*<0.001. NS: No significance.

**Supplementary Fig. 2** (A) CRLF1 staining in PTC tissues and corresponding adjacent normal tissues. (B) CRLF1 staining in liver cancer tissue (Positive control) and corresponding adjacent normal tissues. (C) Immunohistochemical analysis of PTC tissues with control IgG. Cancer cells indicated by yellow arrows. Normal cells indicated by red arrows. CRLF1 can be found in cell cytoplasm.

**Supplementary Fig. 3** (A) Map of the MAPK phosphorylation antibody array. (B) Upregulated markers detected in the assay (fold-change>1.5). POS: Positive control spot; NEG: Negative control spot.

**Supplementary Fig. 4** Immunohistochemical analysis of CRLF1, p-ERK1/2, and p-AKT (Ser473) in nude mice xenografts of CRLF1-expressing and vector-expressing IHH-4 cells. p-ERK1/2 and p-AKT (Ser473) showed cytoplasmic and nuclear localization. CRLF1 can be found in cell cytoplasm.

**Supplementary Fig. 5** (A) The p-STAT3 level was higher in IHH-4-CRLF1 cells than that in IHH-4-Vector cells. (B) IHH-4-CRLF1 was treated with DMSO (0.1% (v/v)) or Stattic (10 μM) for 24h, Western blotting analyses were performed to evaluate the effects of Stattic on phosphorylation levels of STAT3. β-actin was used as a loading control. (C) MTT proliferation assay results demonstrating the inhibition of the proliferation in CRLF1-overexpressing IHH-4 cells treated with Stattic. NS: No significance.
